# Research-based flow cytometry assays for pathogenic assessment in the human B-cell biology of gene variants revealed in the diagnosis of inborn errors of immunity: a Bruton’s tyrosine kinase case-study

**DOI:** 10.3389/fimmu.2023.1095123

**Published:** 2023-04-24

**Authors:** L. del Pino-Molina, L. Y. Bravo Gallego, Y. Soto Serrano, K. Reche Yebra, J. Marty Lobo, B. González Martínez, M. Bravo García-Morato, R. Rodríguez Pena, M. van der Burg, E. López Granados

**Affiliations:** ^1^ Center for Biomedical Network Research on Rare Diseases, Instituto de Salud Carlos III (ISCII)I (CIBERER), Madrid, Spain; ^2^ Lymphocyte Pathophysiology in Immunodeficiencies Group, La Paz Institute for Health Research (IdiPAZ), Madrid, Spain; ^3^ Pediatric Hemato-Oncology Unit, La Paz University Hospital Madrid, Madrid, Spain; ^4^ Clinical Immunology Department, La Paz University Hospital Madrid, Madrid, Spain; ^5^ Department of Pediatrics, Laboratory for Pediatric Immunology, Willem-Alexander Children’s Hospital, Leiden University Medical Centre, Leiden, Netherlands

**Keywords:** BTK - Bruton’s tyrosine kinase, B cells, bone marrow analysis, flow cytometry assays, B-cell signaling

## Abstract

**Introduction:**

Inborn errors of immunity (IEI) are an expanding group of rare diseases whose field has been boosted by next-generation sequencing (NGS), revealing several new entities, accelerating routine diagnoses, expanding the number of atypical presentations and generating uncertainties regarding the pathogenic relevance of several novel variants.

**Methods:**

Research laboratories that diagnose and provide support for IEI require accurate, reproducible and sustainable phenotypic, cellular and molecular functional assays to explore the pathogenic consequences of human leukocyte gene variants and contribute to their assessment. We have implemented a set of advanced flow cytometry-based assays to better dissect human B-cell biology in a translational research laboratory. We illustrate the utility of these techniques for the in-depth characterization of a novel (c.1685G>A, p.R562Q) *de novo* gene variant predicted as probably pathogenic but with no previous insights into the protein and cellular effects, located in the tyrosine kinase domain of the Bruton’s tyrosine kinase (BTK) gene, in an apparently healthy 14-year-old male patient referred to our clinic for an incidental finding of low immunoglobulin (Ig) M levels with no history of recurrent infections.

**Results and discussion:**

A phenotypic analysis of bone marrow (BM) revealed a slightly high percentage of pre-B-I subset in BM, with no blockage at this stage, as typically observed in classical X-linked agammaglobulinemia (XLA) patients. The phenotypic analysis in peripheral blood also revealed reduced absolute numbers of B cells, all pre-germinal center maturation stages, together with reduced but detectable numbers of different memory and plasma cell isotypes. The R562Q variant allows Btk expression and normal activation of anti-IgM-induced phosphorylation of Y551 but diminished autophosphorylation at Y223 after anti IgM and CXCL12 stimulation. Lastly, we explored the potential impact of the variant protein for downstream Btk signaling in B cells. Within the canonical nuclear factor kappa B (NF-κB) activation pathway, normal IκBα degradation occurs after CD40L stimulation in patient and control cells. In contrast, disturbed IκBα degradation and reduced calcium ion (Ca2^+^) influx occurs on anti-IgM stimulation in the patient’s B cells, suggesting an enzymatic impairment of the mutated tyrosine kinase domain.

## 1 Introduction

Inborn errors of immunity (IEI) are an expanding group of rare diseases generally caused by pathogenic variants in genes involved in human immune system generation and function (1), with the largest group being primary antibody deficiencies (PADs), mostly due to intrinsic B-cell defects (2).

Next-generation sequencing (NGS) has boosted the field of IEI, revealing several new entities and accelerating routine diagnoses by the simultaneous analysis of multiple genes (3). NGS has however also expanded the number of atypical presentations, generating uncertainties about the pathogenic relevance of several novel variants (4).

Variant interpretation relies on a careful assessment of the type, allele frequency in the general population, computational predictions, familiar segregation, allelic positioning, previous description and evidence of functional impairment (5).

Research laboratories that provide support to the diagnosis of IEI require accurate, reproducible and sustainable phenotypic, cellular and molecular functional assays to explore the pathogenic consequences of gene variants in human leukocytes and contribute to their assessment. To this end, we have implemented a set of advanced flow cytometry-based assays to better dissect human B-cell biology in a translational research laboratory.

We illustrate the utility of these techniques for the in-depth characterization of a novel (c.1685G>A, p.R562Q) *de novo* gene variant located in the tyrosine kinase domain of the Bruton’s tyrosine kinase (BTK) gene, predicted as being probably pathogenic but with no previous insights into its protein and cellular effects.

Pathogenic variants in BTK lead to X-linked agammaglobulinemia (XLA) or Bruton’s disease (6), a prototypic PAD related to severe impairment in B-cell ontogeny and maturation due to the crucial role of Btk in pre-B/B-cell receptor (BCR) signaling. XLA is predominantly an antibody deficiency and the first monogenic disease described as a primary immunodeficiency (7). The BTK gene encodes the Btk protein that is essential in several crucial steps of B-cell maturation and differentiation (8). The Btk protein consists of 5 domains: pleckstrin homology (PH), Tec homology (TH), Src homology 3 (SH3), Src homology 2 (SH2), and the tyrosine kinase (SH1) (9). Btk is essential in pre-BCR signaling in bone marrow (BM). Btk deficiency therefore blocks the pre-B-I stage in BM. Later in B-cell differentiation in peripheral blood, Btk propagates BCR signaling through intermediate molecules and calcium influx, ultimately leading to NFκB signaling (10, 11).

An apparently healthy 14-year-old male patient was referred to our clinic for an incidental finding of low immunoglobulin (Ig) M levels, detected during his follow-up by pediatric endocrinology for constitutional growth delay. The patient had no history of recurrent or severe infections and reported only immune thrombocytopenia, at the age of 2 years. Routine immunological studies revealed grossly decreased peripheral blood B-cell counts.

**Figure 1:**
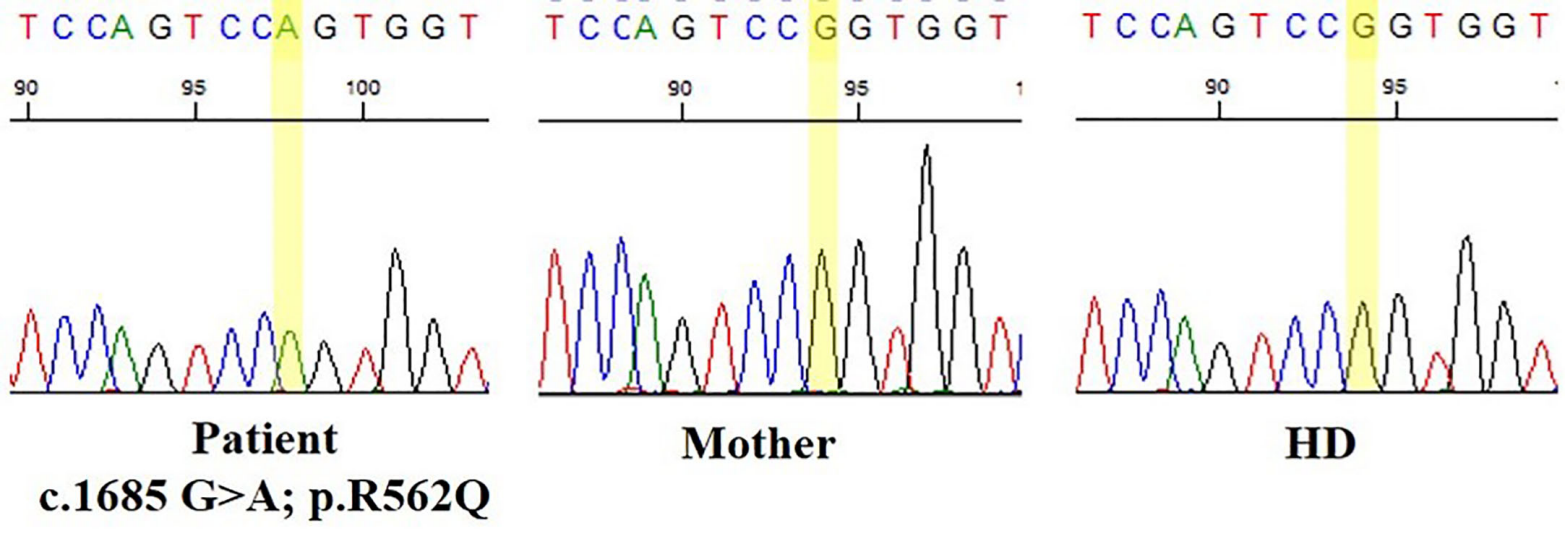
Nucleotide substitution (c.1685G>A, p.R562Q) located in the tyrosine kinase domain of Bruton’s tyrosine kinase (BTK) gene.

After informed consent was provided, NGS genetic studies were performed based on a custom-made IEI panel. Genetic analysis revealed a heterozygous *de novo* variant in the BTK gene (c.1685G>A, p.R562Q) considered probably pathogenic, not previously described and not present in the patient’s parents (**Figure 1**).

In the first visit to the Clinical Immunology Department, profound B-cell lymphopenia was detected, as well as low IgM (27 mg/dL) and normal IgG and IgA levels (1072 mg/dL and 189 mg/dL, respectively), also IgG2 (271 mg/dL) and IgG3 (33,4 mg/dL) were in normal levels. The patient presented normal lymphocyte proliferation with concanavalin A (Con A), pokeweed mitogen (PWM) and OKT3. The patient did not respond to polysaccharide antigen after pneumococcus vaccination with pneumovax^®^ 23, but presented an adequate post-vaccination response to tetanus and diphtheria, also the patient received the SARS-CoV-2 vaccine and generated specific antibodies.

We conducted a series of phenotypic and functional assays in B cells to determine the pathogenicity of this variant in BTK and the functional impact on this patient. To this end, we explored the possible alteration of this crucial protein for early B-cell development by analyzing the phenotype in BM and peripheral blood. To further explore Btk functionality, we selected intracellular readouts measurable by flow cytometry that reflected the strength of homeostatic signaling pathways in resting cells and after activation, such as the protein expression levels of Btk, phospho-Btk and downstream signaling readouts such as intracellular Ca^2+^ influx and IκBα degradation.

## 2 Methods

### 2.1 Samples

Blood samples from the patient and healthy donors (HDs) were collected at La Paz University Hospital after obtaining their informed consent. The study was approved by the hospital’s ethics committee (PI-2833) and adhered to the principles of the Declaration of Helsinki. BM samples from the patient were collected as part of the diagnosis workout of peripheral blood cytopenias. Normal BM samples were taken from the excess material of patients who had undergone a BM biopsy to rule out diseases other than lymphoid PID. The clinical data were obtained from the clinical records updated during routine medical visits for the diagnosis and follow-up at the outpatient clinic of the Clinical Immunology Department.

### 2.2 Flow cytometry phenotype analysis

The patient’s blood samples were processed and stained with the EuroFlow 8-color *PIDOT, Pre-GC B cell* tube and *IgH subclasses* tube (12–15), following the EuroFlow standard operating procedures for staining, instrument set-up and calibration, as previously described (16). The BM samples were processed and stained with a modification of the EuroFlow 10-color *B-cell precursors bone marrow tube* (BCP-BM) (17), adapted to 8 colors in two tubes. Cells were stained for surface membrane (sm) and intracellular markers in two consecutive steps. For the surface staining, the following antibodies were used: IgM-BV510 (MHM-88) CD38-FITC (HIT2) and CD20-PB (2H7) (all from Biolegend; San Diego, CA); CD34-APC (8G12) and IgD-PE (IA6) (all from BD Biosciences); CD19-PC7 (J3-119) (Beckman Coulter); and CD10-APC-C750 (HI10a) (from Cytognos; Salamanca, Spain). The cells were fixed and permeabilized using the Fix&Perm reagent kit (An der Grub, Vienna, Austria) according to the manufacturer’s instructions, and further stained with IgM-PerCPcy5.5 (MHM-88) (Biolegend); TdT-FITC (HT6) (Supertechs Inc., Rockville, MD); and CD79a-PE (HM47) (Beckman Coulter).

B-cell precursor populations were identified as follows: pro B cells (CD19^-^ TdT^+^ CD34^+^), pre-B-I (CD19^+^ cyIgu^-^ IgM^-^), pre-B-II (CD19^+^ cyIgu^+^ IgM^-^), immature (CD19^+^ cyIgu^+^ IgM^+^ IgD^-^) and mature (CD19^+^ IgM^+^ IgD^+^). The latter population was excluded from the calculation of the composition of the precursor B-cell compartment because mature B cells can also result from peripheral contamination. Data acquisition was performed in a FACS Canto II cytometer (BD Biosciences). For the data analysis, a standardized gating strategy was employed to identify all populations, as previously described (14, 15, 17). The analysis was performed with *Infinicyt* software (Cytognos SL, Salamanca, Spain).

### 2.3 Flow cytometry analysis of Btk expression

We performed an intracellular staining protocol to evaluate Btk expression. Whole blood was stained with CD14 BV421, then fixed with 10% formaldehyde and treated with 0.1% Triton X-100. Cells were permeabilized with 50% methanol on ice. The cells were then stained with the following antibodies: CD3 APC (clone UCHT1; BD Biosciences) CD19 PE Cy 7 (clone J3-119; Beckman Coulter), Btk rabbit antibody (clone D3H5), anti-rabbit IgG F (ab´)2 fragment conjugated with Alexa Fluor 488 (Cell Signaling) and the specific isotype control anti-rabbit IgG (Cell Signaling). The mean fluorescence intensity values of Btk were obtained. Samples were acquired in FACS Canto II (BD Biosciences). We performed the data analysis by employing Flow-Jo software (Flow-Jo LLC).

### 2.4 Flow cytometry analysis of BTK phosphorylation

To test Btk functionality, we measured the intracellular content of phospho-Btk (pBtk) at Y551 and Y223 in basal and upon stimulation conditions, based on the manufacturer’s instructions and previous reports, respectively (18, 19). Briefly, peripheral blood mononuclear cells (PBMCs) were obtained after Ficoll density gradient centrifugation (Ficoll-Paque Premium; VWR International, Eurolab). A total of 5x10^5^ PBMCs were resuspended in 100 µL RPMI-1640 supplemented medium (10% FCS, 100 U/mL penicillium, 100 µg/mL streptomycin and 2 mM glutamine). PBMCs were left to rest at 37 °C for 20 min. Cells were stimulated with 20 µg/mL goat F(ab)2 anti-human IgM (µ chain specific) (Southern Biotech) for 30, 60 s and 5 min at 37 °C, while an unstimulated sample was processed in parallel. For pBTK at Y551 cells were then fixed with 4% formaldehyde (Cell Signaling) and permeabilized with 50% methanol on ice. Cells were stained for 1 h following the manufacturer’s instructions with the following antibodies: phospho-Btk (Tyr 551) (E5Y6N) (Cell Signaling), CD19 PE Cy7 (clone J3-119, Beckman Coulter) and CD3 APC (clone UCHT1; BD Biosciences). The specific isotype control, anti-rabbit IgG (Cell Signaling) was added at the same concentration as the specific antibody. Lastly, the cells were stained with the secondary antibody anti-rabbit IgG (H+L), F (ab´)2 fragment conjugated with Alexa Fluor 488 for 30 min.

For pBTK at Y223 PBMCs were stained with IgD BV605 and CD27 BV421 (BD Bioscience) and then stimulated with 20 µg/mL goat F(ab)2 anti-human IgM (µ chain specific) (Southern Biotech) for 2 and 5 min or with 1 µg/ml CXCL12 (R&D) for 2 min at 37 °C. Cells then were treated with fixation/permeabilization buffer (eBioscience), followed by wash buffer and permeabilization buffer (eBioscience) and stained with CD19 FITC, CD3 APC, pBTK PE (Y223) and isotype PE control (BD Biosciences).

Responsiveness to BCR stimulation was calculated as the ratio of phospho-Btk in stimulated cells at different times (30 s, 60 s, 2 min or 5 min) to phospho-Btk in unstimulated cells and expressed as the fold increase. Samples were acquired in Dx Flex (Beckman Coulter) and analyzed using Flow Jo software (Flow-Jo LLC).

### 2.5 Flow cytometry analysis of calcium flux

Intracellular Ca^2+^ influx was measured using FuraRed (FuraRed, cell permeant; Thermo Fisher Scientific). A total of 1x10^6^ PBMCs from the patient and HDs were incubated with 1 μM Fura Red-AM in loading buffer (Hank’s balanced salt solution medium supplemented with 5% fetal calf serum) at 30 °C for 30 min in the dark (20). To analyze the B cells, during the final 10 min the cells were stained with CD19 APC (BD Biosciences). Cells were then washed and resuspended in loading buffer and then warmed to 37 °C for 5 min prior to acquisition. A baseline was recorded for 60 s, followed by stimulation of the BCR with 35 μg/mL anti-IgM (F(ab)2 anti-Human IgM; Southern Biotech). The cells were then stimulated with 5 μg/mL ionomycin (Sigma) to measure maximum Ca^2+^ signaling. Data were acquired with a FACS Canto II flow cytometer (BD Biosciences), and data analysis was performed with the use of FlowJo software (Flow-Jo LLC).

### 2.6 Flow cytometry analysis of IκBαdegradation

The intracellular content of IκBα was determined by using intracellular flow cytometry in baseline conditions and degradation upon stimulation. PBMCs were left to rest at 37 °C for 30 min and simultaneously stained with anti-CD27 BV421 (clone M-T271; BD Biosciences). Cells were stimulated at 37 °C with 20 µg/mL goat F(ab)2 anti-human IgM (µ chain specific) (Southern Biotech) for 90 min, 1 µg/mL MegaCD40L soluble human recombinant protein (Enzo Life Sciences) for 15 min, 200 ng/mL of phorbol 12-myristate 13-acetate (PMA; Sigma-Aldrich) for 15 and 90 min, while in parallel an unstimulated sample was processed for each time point. Cells were then fixed with 4% formaldehyde (Cell Signaling) and permeabilized with 50% methanol on ice. Cells were stained for 60 min with the following antibodies: IκBα (L35A5) Alexa Fluor 488 (Cell Signaling), CD19 PE Cy7 (clone J3-119; Beckman Coulter), CD3 APC (clone UCHT1; BD Biosciences) and IgD PE (Southern Biotech). The specific isotype control anti-mouse IgG1 (Cell Signaling) was added at the same concentration as the specific antibody. Responsiveness to stimulation was calculated as the ratio of IκBα in stimulated cells at various times (30 s, 60 s and 5 min) to IκBα in unstimulated cells and expressed as the fold increase. Data were acquired on a DxFlex flow cytometer (Beckman Coulter), and data analysis was performed with the use of FlowJo software (FlowJo, LLC).

### 2.7 Statistics

The data analysis was performed using GraphPad Prism version 9.0 software (San Diego, CA, USA). We determined the statistical differences between the patient and controls by applying the nonparametric Mann-Whitney test. Differences were considered statistically significant for p-values of * <0.05, ** <0.01 and *** <0.001.

## 3 Results

### 3.1 Phenotypic analysis of the B-cell compartment in bone marrow and peripheral blood

**Figure 2:**
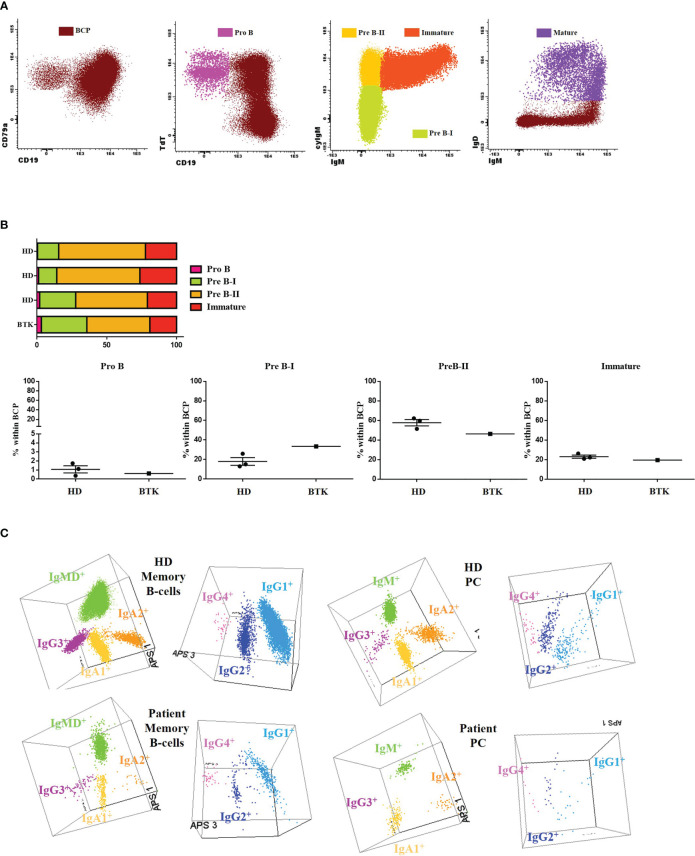
B-cell subsets: **(A)** The results of the IgH isotype and subclasses distribution in memory B cells (MBC) and plasma cells (PC) analyzed by flow cytometry in the patient and a representative healthy donor (HD). Representation on a balanced 3-dimension (3D) automated population separator (APS) diagram, constructed using the first three principal components (PC1 to PC3) derived from PC analysis (PCA) performed with the *Infinicyt* software. **(B)** Gating strategy used for classifying B-cell precursors (BCP) (CD79a^+^) and then dissecting into different subsets: Pro B (CD19^-^ TdT^+^ CD34^+^), Pre-B-I (CD19^+^ cyIgu^-^ IgM^-^), Pre-B-II (CD19^+^ cyIgu^+^ IgM^-^), immature (CD19^+^ cyIgu^+^ IgM^+^ IgD^-^) and mature (CD19^+^ IgM^+^ IgD^+^). **(C)** BCP subset distribution in the patient and 3 age-matched HDs.

The patient’s clinical evaluation included a complete analysis of the phenotypic maturation from BM to peripheral blood due to B-cell lymphopenia. The patient presented a roughly similar distribution of pro-B, Pre-B-I, Pre-B-II and immature B cell compartments but rather high percentages of Pre-B-I cells when compared with the age-matched (HDs) (**Figures 2A, B**).

Severely reduced absolute B-cell counts were observed in peripheral blood when compared with the age-matched HDs analyzed with the *PIDOT tube*-EuroFlow (**Table 1**). The patient presented total T-cell counts, including naïve, central memory, effector memory and effector CD4^+^ and CD8^+^ T cells, TCRγδ^+^ T cells and NK cells in normal ranges for his age. Other leukocyte populations (eosinophils, neutrophils, monocytes, monocytes CD16^+^, plasmacytoid dendritic cells and basophils) were also in normal ranges.

**Table 1:** Distribution of distinct peripheral populations analyzed with the EuroFlow Primary Immunodeficiency Orientation Tube (PIDOT) vs. age-matched reference values.

	Patient	Age reference values
Lymphocytes	2801	(1012-4792)
B-cells	**27.5**	(59.5-1194)
Pre-GC B-cells	**21.6**	(22.1-1040)
Post-GC B-cells MBC	**6**	(23-276)
Unswitched MBC	**2.4**	(9-155)
Switched MBC	**3.5**	(12-182)
T-cells	2399	(564-3477)
CD4^+^	1730	(361-1900)
Naive	1394	(150-1484)
Central memory	287	(121-885)
Effector memory	46.2	(100-500)
Effector TD	2.5	(0-87)
CD8^+^	558	(160-1189)
Naive	385	(33.1-986)
Central memory	105	(54-456)
Effector memory	16.3	(2-69.2)
Effector TD CD27^+^	17.6	(0-144)
Effector TD CD27^-^	34	(1-325)
CD4^-^CD8^-^ TCRγδ^+^	86.8	(11-470)
CD4^-^CD8^-^ TCRγδ^-^	21.8	(6-80)
NK cells	375	(81-1348)
Eosinophils	62.1	(15-1077)
Neutrophils	1545	(811-9132)
Monocytes	682	(124-1063)
Non-classical monocytes (CD16^++^)	108	(9-177)
Plasmacytoid dentritic cells	23.6	(4-64)
Basophils	64.8	(1-132)

Results expressed as absolute cell counts per µL of peripheral blood. Normal reference values are expressed as minimum and maximum values previously reported for age-matched healthy donors12. In bold altered cell numbers.

We then explored in detail the peripheral blood B-cell compartment through the *Pre GC-B cell* and *IgH subclasses-B cell* tubes (**Table 2**). The percentage distribution of main B-cell subsets was almost normal (58% naïve B-cells, 8% IgMD^+^ memory B cells (MBCs) and 10% IgMD^-^ switched MBCs). However, all pre-germinal center maturation stages, from immature/transitional (CD5^+^ CD38^het^ CD21^+^ CD24^+^) to naïve B cells (all of them CD21^+^ CD24^+^) were present, albeit with low absolute counts. The analysis of the post-germinal B-cell compartment at the specific isotype level revealed detectable but reduced absolute counts of IgMD^+^, IgG1^+^, IgG2^+^, IgG3^+^, IgA1^+^ and IgA2^+^ MBCs and normal numbers of IgG4^+^ MBCs. The plasma cell diversification was partial, with normal absolute counts of IgM^+^ cells, reduced but detectable IgG1^+^, IgG2^+^, IgA1^+^ and IgA2^+^ cell counts and absent IgG3^+^ and IgG4^+^ cells (undetectable <0.01 cells/µL). **Figure 2C** shows a comparison of MBC and plasma cell (PC) subsets expressing different immunoglobulin subclasses in 3D automated population separator (APS) diagrams for the patient and a representative HD.

**Table 2:** Distribution of different subsets of blood B-cells, including MBC and plasma cells subsets expressing different immunoglobulin-subclasses with the EuroFlow pre-Germinal Center B-cell tube (pre-GC) and immunoglobulin isotype B-cell tube (IgH isotype B-cell tube) vs. age-matched reference values.

B-cell population	Patient	N° of B cells/µl (range)
Total B-cells	**16.6**	206 (57-402)
Immature/transitionals	**3.1**	27 (5.7-126)
CD5^-^ CD38^++^ CD21^het^ CD24 ^++^	1.5	0.89 (0.13-4)
CD5^+^ CD38^+/++^ CD21^het^ CD24^++^	0.9	5.60 (1.1-35)
CD5^+^ CD38^het^ CD21^+^ CD24^+^	**0.7**	17 (4-94)
Mature naive B-cells	**9.7**	85 (24-203)
CD21^+^ CD24^+^	**9.7**	84 (23-201)
CD21^-^ CD24^++^	–	0.74 (0.02-3.7)
CD21^-^ CD24^-^	**-**	0.4 (0-0.14)
Memory B-cells	**3.2**	72 (19-159)
Memory IgMD^+^	**1.5**	32 (7.6-88)
Memory IgMD^-^	**1.7**	35 (11-90)
IgG1^+^	**0.6**	18 (7-42)
IgG2^+^	**0.2**	3 (0.7-10)
IgG3^+^	**0.12**	3 (1.1-8.3)
IgG4^+^	0.07	0.2 (<0.01-2.9)
IgA1^+^	**0.46**	9 (2.9-21)
IgA2^+^	**0.04**	4.1 (1.2-18)
Plasma cells	**0.92**	8.5 (1.3-27)
IgM^+^	0.26	0.8 (0.2-5.7)
IgG1^+^	**0.03**	1.1 (0.1-4.8)
IgG2^+^	**0.04**	0.5 (0.08-0.8)
IgG3^+^	Und*	0.08 (<0.01-0.4)
IgG4^+^	Und*	<0.01 (<0.01-0.2)
IgA1^+^	**0.27**	3.1 (0.5-14)
IgA2^+^	**0.07**	1 (0.3-3.5)

Results expressed as absolute cell counts per µL of blood. Normal reference values are expressed as (range: 5th and 95th percentile values) previously reported for healthy donors13, 14. In bold altered cell numbers. *Und (undetectable) when less than 0.01 cells/ul or less than 10 events recorded.

**Figure 3:**
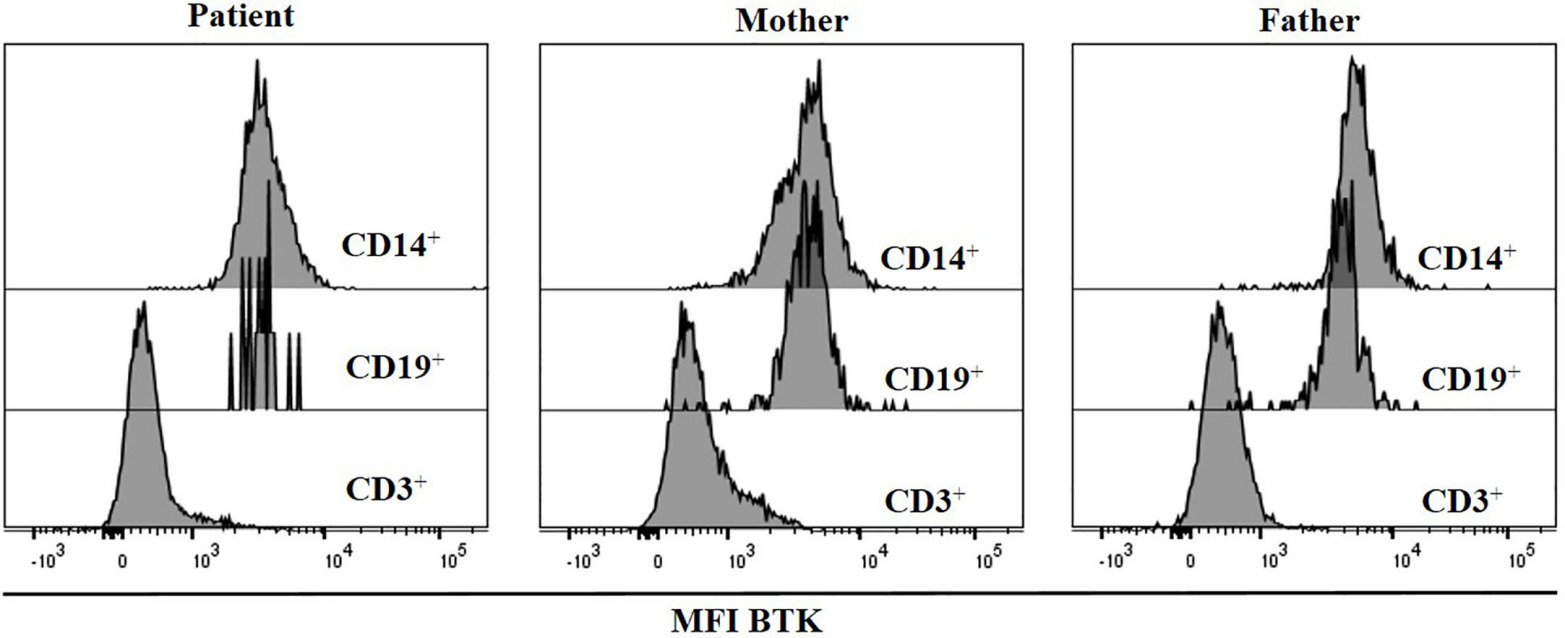
BTK expression was measured by flow cytometry. Histograms show BTK expression in CD19^+^, CD3^+^ and monocytes, in the patient and his parents.

We also measured Btk expression by flow cytometry; the R562Q variant does not preclude intracellular protein expression, with the patient’s B cells and monocytes revealing comparable MFI to those of the HD. Complete absence of expression in T cells was used as negative control for the antibody staining (**Figure 3**).

### 3.2 Intracellular signaling

**Figure 4:**
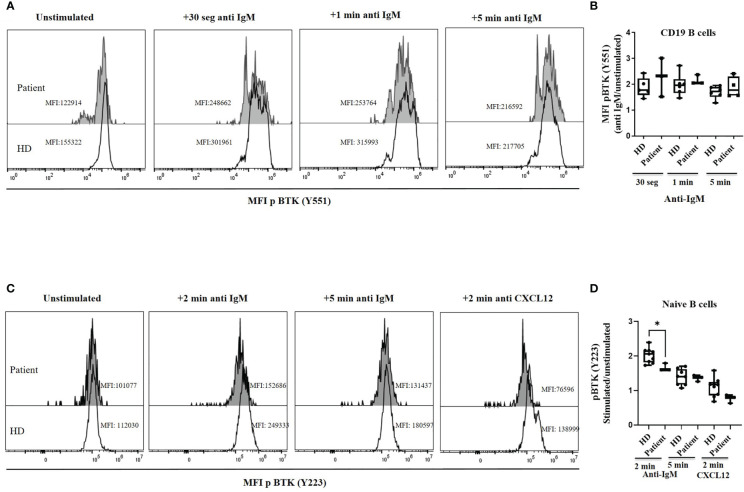
BTK phosphorylation. **(A)** Histograms of a representative healthy donor (HD) and patient, showing the median of mean fluorescence intensity (MFI) of p Btk (Y551) in CD19^+^ B cells in unstimulated and BCR-mediated activation conditions with anti-IgM. **(B)** pBtk (Y551) in B cells expressed as the induced MFI at 30 s, 60 s and 5 min activation with anti-IgM compared with the unstimulated cells in B cells from HD and patient. The patient was analyzed twice in duplicates. **(C)** Histograms of a representative healthy donor (HD) and patient, showing median mean fluorescence intensity (MFI) of pBtk (Y223) in naïve-B cells in unstimulated and BCR-mediated activation conditions with anti-IgM or CXCR4 activation with CXCL12. **(D)** pBtk (Y223) in naïve-B cells expressed as the induced MFI at 2 and 5 min activation with anti-IgM or after 2 min with CXCL12 compared with the unstimulated cells from HD and patient. *<0.05.

We studied BTK functionality upstream of the variant (p.R562Q) analyzing the Btk phosphorylation levels at Y551 found in patient´s cells. BCR activation induced Tyr551 phosphorylation, which in turn enhances the enzymatic activity of the protein, is similar in the patient’s B cells and healthy controls at 30 s, 60 s and 5 min upon anti-IgM BCR activation (**Figures 4A, B**). The second event of phosphorylation for a complete Btk activation is an autophosphorylation at Y223. This was analyzed at different time points of stimulation in B cells with anti IgM in HD, resulting in a maximum of pBtk at Y223 after 2 minutes and decay after 5 minutes of BCR stimulation. The pBtk induction at Y223 was diminished in total B cells (data not shown) and naïve B-cells in the patient in comparison with HD after 2 minutes of stimulation. The patient´s naïve B-cells cannot reach this peak after 2 minutes of stimulation with anti IgM, but presented levels of pBtk induction after 5 minutes similar to HD (**Figures 4C, D**). CXCL12 stimulation produces a slight increase in pBTK levels at Y223 in total B cells and naïve-B cells from HD, which once again is reduced in the patient cells after 2 minutes of CXCR4-CXCL12 signaling (**Figures 4C, D**).

**Figure 5:**
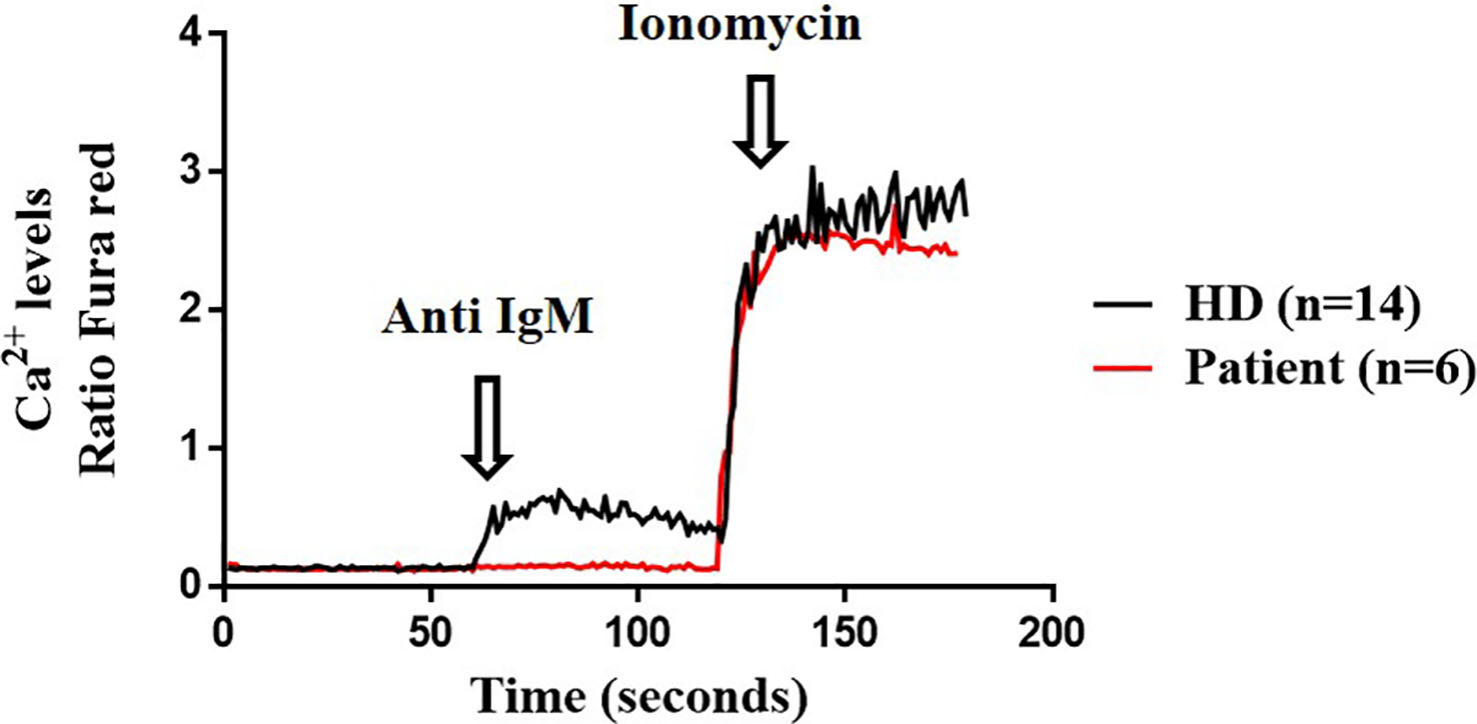
Calcium influx. Cytosolic calcium ion (Ca^2+^) levels at baseline and induced by anti-IgM and ionomycin in B cells from the patient (twice in triplicates) and healthy donor (HD) (n=14).

Lastly, we explored the potential impact of the variant protein for downstream Btk signaling in B cells, by analyzing activation read-outs such as Ca^2+^ flux and IκBα degradation. Ca^2+^ influx in B cells was run in triplicates in two separate times. The R562Q variant decreases the Ca^2+^ influx upon anti-IgM stimulation in the patient’s B cells compared with the HD but reached the maximum Ca^2+^ signaling when stimulated with ionomycin as the positive control, similar to the HD (**Figure 5**).

**Figure 6:**
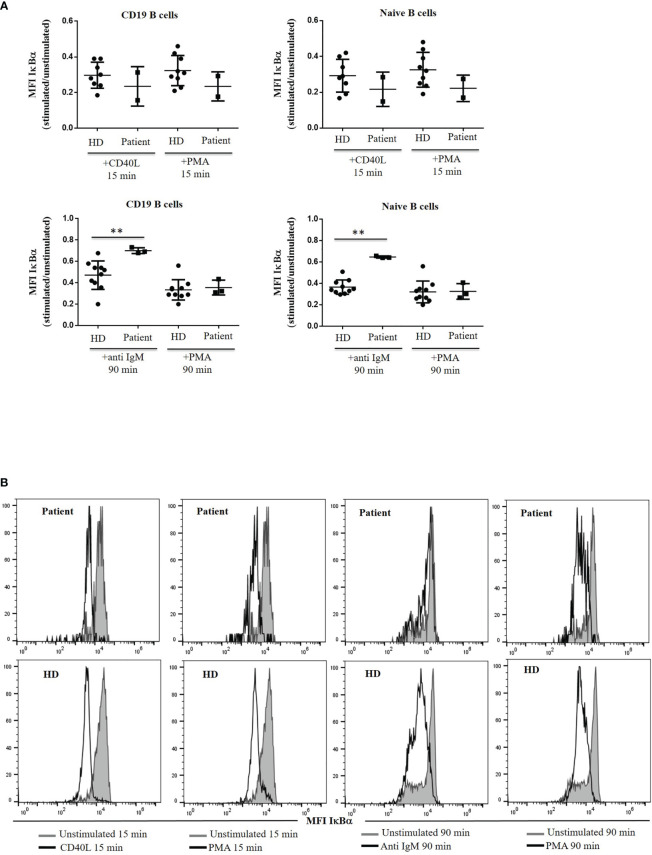
IκBα degradation. **(A)** IκBα levels are expressed as the induced MFI at 15 or 90 min of activation with CD40L, anti IgM and PMA, compared with the unstimulated cells in CD19^+^ and naïve B cells from the patient and healthy donor (HD). **(B)** Histograms of representative HD and patient showing mean fluorescence intensity (MFI) of IκBα in unstimulated and stimulated CD19^+^ and naïve B cells with CD40L, anti IgM and PMA from the patient and HD. **<0.01.

Within the canonical NF-κB activation pathway, we analysed IκBα degradation as a readout, we observed similar ratios at 15 min after CD40L stimulation in the patient’s B cells and the control cells. In contrast, clearly defective BCR downstream signaling was evidenced from the significantly reduced folds of anti-IgM-induced IκBα degradation at 90 min (**Figure 6**).

## 4 Discussion

This manuscript presents a broad set of assays that could be applied to explore the pathogenic consequences of gene sequence variants in Btk or other functional related proteins. We validated their utility in revealing the pathogenicity of a novel, clinically less severe and immunologically less disturbing R562Q BTK variant.

Classical male XLA patients present severe agammaglobulinemia due to the severe reduction or absence of peripheral B cells, and experience frequent infections (7). However, BTK variants associated with atypical XLA case presentations have been reported, in patients with normal: IgG levels (21), selective IgM deficiency (22) with a leaky phenotype (in a Japanese family) (23), a diagnosis in adulthood but with a history of recurrent infections (24) and with impaired polysaccharide responsiveness without marked hypogammaglobulinemia (25). Although the gene variant present in our patient (R562Q) had not been previously described, other variants have been reported in the same residue as being probably pathogenic (R562W, R562L, R562P) (26–30). One of these variants was present in a patient with low IgG levels but normal IgM and IgA levels (27). R562 is located in the Btk kinase domain in close proximity to the catalytic site and is involved in substrate binding and together with A582 is implicated in the interaction with the invariant W563 chain (31). These three residues are highly conserved in Btk, and mutations in the surrounding residues might interfere with their interaction and the catalytic loop (32, 33). R562W amino acid substitution has been associated with a delayed symptom onset (32). Here, we present a patient with a leaky BTK mutation, a *de novo* variant in an individual with no previous family history of the disease and with no history of recurrent infections and a casual finding of reduced IgM levels.

Btk plays a critical role in signal transduction in relevant signaling pathways implicated in B-cell survival, activation, proliferation and differentiation. BCR signaling activates Btk by Lyn and Syk through Y551 phosphorylation, and then a second event by auto-phosphorylation at Y223 is required for a complete Btk activation (34). Btk is also implicated in B-cell migration and homing to lymph nodes through its activation by chemokine receptors such as CXCR4 and its ligand CXCL12 (11).

A severe block in the maturation of early BM B-cell precursors is commonly seen in XLA patients, typically at the Pre-B-I to Pre-B-II transition. Our patient presented a normal distribution of BCP in BM but a slight expansion of the pre-B-I compartment. Classical XLA patients present the blockage in Pre-B-I with a severe reduction of immature B cells in BM and almost absent B cells in peripheral blood (17, 35). Our patient presented a severe reduction in B cells in peripheral blood in terms of absolute counts, but the distribution of B-cell subsets in percentages were almost in the normal range, and the patient was even able to produce MBCs and PCs. The detection of low MBC and PC counts expressing IgG1 to IgG4 and IgA1 to IgA2 showed that the patient retained the ability for class-switching (36), conferring protection against a wide variety of pathogens. The patient is currently free of infection, presents sustained specific antibody responses upon protein vaccination, including SARS-CoV-2 vaccine and is not undergoing immunoglobulin replacement therapy but under a close monitoring to detect deterioration of the diversity of the B cell compartment or an increased susceptibility to respiratory infections.

The R562Q variant does not preclude intracellular protein expression. The patient’s B cells and monocytes were comparable to those of the healthy controls, as previously reported for R562P (28) and certain other Btk mutations (6).

Btk plays a key role in B-cell development and maturation, participating in the intracellular pre-BCR and BCR-induced Ca^2+^ influx, being the latest critical step for several proliferative responses at the Pre-B-I and several other peripheral stages. The R562Q variant decreases Ca^2+^ influx on anti-IgM stimulation in the patient’s B cells, suggesting an enzymatic impairment of the mutated tyrosine kinase domain, as previously reported in other hypomorphic mutations (25) with Btk-deficient B-cells that exhibit defective anti-IgM calcium mobilization but normal induction of PLCγ2 tyrosine phosphorylation (37). Similar findings in point mutations from kinase, SH and PH domains in DT40 cells have shown that Btk deficiency causes a reduction in hydrogen peroxide levels induced by the calcium response, and this altered calcium signaling causes the suppression of IP3 production and phospholipase PLCγ2 (38). Also, DT40 cells transfected with several point mutations that exhibited altered Ca^2+^ influx upon IgM engagement were restored when transfected with wildtype Btk (39). Several studies have described the relevance of Btk in platelets, which are activated after thrombin stimulation leading to Btk phosphorylation, an analogous process to what occurs in B cells and BCR activation. In fact, calcium mobilization is reduced in Btk-deficient platelets (40).

Btk phosphorylation at Tyr551was similar in the patient’s B cells and healthy controls at 30 s, 60 s and 5 min of anti-IgM BCR activation. BCR signaling therefore appears to be unaltered at this point with residual Btk function as found in other Btk variants in humans (25). Similar findings have been reported in an X-linked immunodeficient (*Xid)* mutant where Btk can be tyrosine-phosphorylated upon BCR stimulation as wild-type Btk (41).

However, the second event for a complete BTK activation is an autophosphorylation of Btk at Y223 (42). Here, we found reduced pBtk levels after anti IgM and CXCL12 in patient´s naïve B-cells. The R562Q variant might produce a delay in Btk autophosphorylation, since patient´s naïve B-cells cannot reach the maximum peak of pBtk induction at 2 min observed in HD, but after 5 min of stimulation with anti IgM the phosphorylation levels at Y223 are comparable to those of HD. Several previous studies have shown absent to reduced phosphorylation of pBtk at Y223 after BCR stimulation depending on the variant analyzed (39). Vihinen et al, detected defective autophosphorylation at Y223 in the missense mutation R562P (28, 33). The CXCR4-CXCL12 axis activates Btk (11), that promotes the signal transduction for B-cell migration and homing to lymph nodes (43), and our results showed that the R562Q variant might also affect this signaling pathway.

Lastly, we explored the potential impact of the variant protein for downstream Btk signaling in B cells. BTK is the nexus between the BCR and NFκB. BTK together with PI3K, is essential for NFκB activation, mediated by IκBα degradation to ultimately promote NFκB translocation to the nucleus (41). We tested the NFκB signaling by analyzing IκBα degradation with CD40L, anti IgM and PMA as the positive control. Within the canonical NFκB activation pathway and using IκBα degradation as a readout, we observed similar ratios at 15 min after CD40L stimulation in the patient’s and control’s cells, as well as with PMA. In contrast, clearly defective BCR downstream signaling was observed due to the significantly reduced folds of anti-IgM-induced IκBα degradation at 90 min. Our results with the R562Q variant are similar to those with the *Xid* mutant Btk with conserved tyrosine phosphorylation on BCR stimulation as wild-type Btk but with altered IκBα degradation (41). Disturbed IκBα degradation with anti-IgM stimulation in *Xid* mice with Btk deficiency can be rescued when activated with PMA and ionomycin because its signaling is independent of Btk (44). Other studies have also reported conserved NFκB nuclear translocation with CD40L but diminished with anti-IgM in *Xid* mice with R28C mutation in Btk (45).

The novel R562Q variant present in a 14-year-old patient produced a minimal block in Pre-B-I stage in BM that allows for further B-cell differentiation in BM and peripheral blood with a reduced but quite diverse repertoire of MBCs and PCs, with normal Btk expression in B cells and monocytes and correct Btk phosphorylation at Y551 following BCR stimulation with anti-IgM. However, downstream BCR signaling is altered in terms of IκBα degradation and reduced Ca^2+^ influx upon IgM engagement, suggesting the impairment of the mutated tyrosine kinase domain, which might be critical for several proliferative responses at the Pre-B-I and several other peripheral stages.

The advent of NGS has expanded enormously the diagnostic capability of IEI. The implementation of advanced phenotypic and functional assays for flow cytometry in diagnostic and supportive translational research laboratories is also mandatory, as exemplified by the assessment of the functional impact of this novel Btk R562Q variant in a patient with B cell functional and phenotypic impairments but absent clinical expressivity so far in terms of increased susceptibility to infections.

In addition, the implementation of these methods to better understand B-cell biology will be useful in new clinical scenarios, such as patients treated with Btk inhibitors for malignancies and autoimmune diseases (46).

## Data availability statement

The original contributions presented in the study are included in the article/Supplementary Material. Further inquiries can be directed to the corresponding authors.

## Ethics statement

The studies involving human participants were reviewed and approved by La Paz University Hospital ethics committee (PI-2833). Written informed consent to participate in this study was provided by the participants’ legal guardian/next of kin.

## Author contributions

LPM, ELG and YB conceived the project and designed the experiments. LPM, KR, YS and JM performed the experiments. LPM and ELG wrote the manuscript. All authors contributed to the article and approved the submitted version.
